# Immune Functions of Erythrocytes in Osteichthyes

**DOI:** 10.3389/fimmu.2020.01914

**Published:** 2020-09-15

**Authors:** Michał Stosik, Beata Tokarz-Deptuła, Jakub Deptuła, Wiesław Deptuła

**Affiliations:** ^1^Faculty of Biological Sciences, Institute of Biological Sciences, University of Zielona Góra Góra, Poland; ^2^Institute of Biology, University of Szczecin, Szczecin, Poland; ^3^International Hereditary Cancer Center, Pomeranian Medical University, Szczecin, Poland; ^4^Institute of Veterinary Medicine, Nicolaus Copernicus University, Torun, Poland

**Keywords:** fish, erythrocytes, immunity, viruses, bacteria, fungi

## Abstract

Red blood cells (RBCs)—erythrocytes—of Osteichthyes are primarily known for their involvement in the process of gas exchange and respiration. Currently, physiological properties of RCBs in fish should also include their ability to participate in defense processes as part of the innate and adaptive immune mechanisms. In response to viruses, bacteria, and fungi or recombinant nanoparticles, they can modulate expression of genes responsible for immune reactions, influence activity of leukocytes, and produce cytokines, antimicrobial peptides, and paracrine intercellular signaling molecules. Via the complement system (CR1 receptor) and owing to their phagocytic properties (erythrophagocytosis), RBCs of Osteichthyes can eliminate pathogens. In addition, they are probably involved in the immune response as antigen-presenting cells via major histocompatibility complex class II antigens.

## Introduction

Red blood cells (RBCs)—erythrocytes—are the most abundant blood cells in Osteichthyes. Their number, as per the species and biological activity of fish, range between 0.5 and 1.5 × 10^6^/mm^3^ and 3.0 and 4.2 × 10^6^/mm^3^ (1). The cells have a shape of oval, flattened, and biconvex discs. They are characterized by a nucleus and organelles in the cytoplasm, i.e., endoplasmic reticulum, ribosomes, Golgi apparatus, and mitochondria (2, 3). RBCs are primarily known for their involvement in the process of gas exchange and respiration (4). Currently, physiological properties of erythrocytes in fish also include their ability to participate in immune processes. The immune functions of nucleated red blood cells in Osteichthyes, but also in amphibians, reptiles, and birds, are increasingly better explored and gain their importance as the cells supporting the mechanisms of innate and adaptive immunity in these animals (5). In response to stimulating factors, e.g., IB^TNFα^ and IB^frg16G−VHSV^ nanoparticles, erythrocytes of Osteichthyes can modulate expression of genes responsible for immune reactions (5, 6) and leukocyte activity as well as produce cytokines, such as interleukin-1β (IL-1β), IL-8, tumor necrosis factor-α (TNF-α), interferon γ (IFNγ), antimicrobial peptides, e.g., β-defensin (BD1) and Nk-lysin (Nkl), and paracrine intercellular signaling molecules (2, 3, 5, 7–11). In Osteichthyes, RBCs can also contribute to elimination of pathogens/antigens, bound in the immune complexes containing the complement, through their recognizing and binding by the complement receptor 1 (CR1) (12). These cells show phagocytic properties (erythrophagocytosis) (12, 13), are capable of reacting with various pathogen-associated molecular patterns via pattern recognition receptors, e.g., peptidoglycan recognition proteins, as well as Toll-like receptors (TLRs) and retinoic acid–inducible gene-I-like receptors (RIG-I-like receptors) (2, 5, 14). Moreover, erythrocytes of Osteichthyes are probably involved in the immune response as the antigen-presenting cells (APCs), via major histocompatibility complex (MHC) class II antigens, and they can contribute to formation of immune synapse with T cells and NK (natural killer) cells (15). In Osteichthyes, erythrocytes have been shown to participate in anti-infection immunity against viruses (4, 6, 9–11, 16, 17), bacteria (13), and fungi (18).

It is worth noting that similar properties, although to a lesser extent and slightly less expressed, are manifested by mammalian (including human) erythrocytes (2, 7, 13, 14, 19–21). It is known that erythrocytes in these organisms mostly evolved in the way that ensures coverage of the body oxygen demand, losing their nucleus and other cellular organelles, such as mitochondria and Golgi apparatus, and therefore, losing the ability to synthesize biologically active molecules in the immune response. Regardless of that, they function as important cells concerning activation and/or stabilization of the innate immune mechanisms. They have immunomodulating properties manifested by the ability to bind chemokines, which ensures a well-balanced/safe level of immune response activation. Another property of mammalian erythrocytes is their ability to bind DNA derived from mitochondria (mtDNA) and pathogens as well as to generate reactive oxygen species (ROS) by hemoglobin released to the extracellular pace (2, 7, 13, 14, 19–21).

## Antiviral Activity of Erythrocytes in Fish

The immune response of erythrocytes in Osteichthyes varies and depends on different viral antigens, referring both to the scope and strength of effects. Viral infections in these animals, studied in terms of erythrocyte immune functions, include infections caused by viral hemorrhagic septicemia virus (VHSV) (4, 6, 10), rock bream iridovirus (RBIV) (16), piscine orthoreovirus (PRV) (17), infectious salmon anemia virus (ISAV) (11), and infectious pancreatic necrosis virus (IPNV) (9). Immune activity of erythrocytes in rainbow trout (*Oncorhynchus mykiss*) has been also demonstrated in the response to polyinosinic:polycytidylic acid (poly I:C) immunostimulation and it was manifested by, among others, expression of TLR, IFN-α-encoding genes, and mediators recruiting immune cells, such as C-C motif chemokine ligand 4 (CCL4) (2).

### Viral Hemorrhagic Septicemia Virus

The VHSV, belonging to the family *Rhabdoviridae*, genus *Novirhabdovirus*, is characterized by a negative-sense single-stranded RNA (ssRNA) genome which encodes five structural proteins (L, G—the main antigenic protein of the virus, N, P, and M). The VHVS causes viral hemorrhagic septicemia (VHS) which occurs in many fish species, not only in the salmonids. It is considered that the causes of VHSV-infected fish mortality are extensive focal degeneration and necrosis of, e.g., kidneys and hematopoietic tissue (The ICTV Report, https://talk.ictvonline.org/ictv-reports/ictv_online_report/).

*In vitro* and *in vivo* studies have revealed (6) that erythrocytes of the rainbow trout (*O. mykiss*), in response to nanostructured recombinant proteins TNFα (IB^TNFα^) and the fragment 16 of VHSV glycoprotein G (IB^frg16G−VHSV^), can modulate expression of immune genes. These cells bind and capture IB^TNFα^ and IB^frg16G−VHSV^ nanoparticles at the level 5–7% of RBCs (RBCs^+^) and in the case of a higher concentration of the nanoparticles, the percent amount of RBCs^+^ increases as high as up to 17%. However, the process of nanoparticle binding and capturing was always more related to IB^TNFα^ (6). The immune response of *in vitro* IB^TNFα^-induced erythrocytes was manifested by decreased expression of the genes *mhc I* (major histocompatibility class I, MHC I); *cd83* (cluster of differentiation 83, CD83), a glycoprotein considered a marker of dendritic cells; and the *gstp1* (glutathione S-transferase pi 1, GST-Pi1), an oxidative stress–related enzyme (6). The IB^frg16G−VHSV^-induced erythrocytes responded with increased expression of the *trx* (thioredoxin, TXN, currently *txn* according to NCBI) which, similarly to the *gstp1*, is an oxidative stress–related gene. In the case of other genes studied in the rainbow trout (*O. mykiss*), such as the *mhc II, tlr3* (toll-like receptor 3, TLR3), *tlr9* (toll-like receptor 9, TLR9), and *il15* (interleukin 15, IL15), no expression changes were observed (6). Analogical *in vivo* studies revealed that the IB^TNFα^-induced erythrocytes responded with decreased expression of the *cd83* (as in the *in vitro* studies) and the *mhc II, tlr9, ifn1, il1*β, *il2, nkef* (natural killer cell enhancement factor, NKEF) as well as with higher expression of the *il6* (6). In the same environment, the IB^frg16G−VHSV^ nanoparticles stimulated increased expression of the *il2, il6, nkef*, *tlr3, cd83, mhc II*, and *mx* (interferon-induced GTP-binding protein Mx) (6). According to the findings (6), IB^TNFα^ and IB^frg16G−VHSV^-induced RBCs of the rainbow trout (*O. mykiss*) stimulate the innate immune response, similarly to anterior kidney macrophages of the rainbow trout (*O. mykiss*) and zebrafish (*Danio rerio*) liver cells, in response to IB^TNFα^ and IB^CCL4^ (22) as well as IB^VP2−IPNV^, IB^G−frg16−VHSV^, and IB^C−VNNV^ (viral nervous necrosis virus, VNNV) (23). In addition, increased expression of the *cd83* and *mhc II* in the rainbow trout (*O. mykiss*) (6), induced by IB^frg16G−VHSV^, suggests a probable ability of these cells to present antigens to the T cells, so they demonstrate the properties of APCs. However, in the view of these data, reports concerning the concept of non-professional APCs presence should be considered; for which there is no evidence of their ability to present antigen to T lymphocytes (6). Regardless of that, it should be noted that with relatively small amounts of RBCs participating in the response (RBCs-−5–7%, macrophages-−40% and more), these cells significantly sooner than macrophages achieve the maximum engulfing level in phagocytosis and the maximum influence on other immune cells, which is of some importance regarding their amount in 1 mm^3^ (6, 24). Owing to the demonstrated scope and type of RBCs' immune activity in Osteichthyes, they are currently considered mediators of immune cells (21, 25). RBCs of Osteichthyes are capable of responding to viral infections (1, 4, 6, 9, 10, 16, 17, 25), producing cytokines (2, 5, 26) and endocytosis of nanoparticles and viral pathogens (11, 15).

Among the proteome proteins of the rainbow trout (*O. mykiss*) VHSV-exposed erythrocytes, the presence of IFIT5 (interferon-induced proteins with tetratricopeptide repeats, IFIT) with antiviral activity has been observed (4). A correlation between the highest level of IFIT5 gene expression and decreased VHSV replication as well as significantly increased VHSV replication in the RBCs of these fish following the ifit5 silencing have been observed. Moreover, in the proximity ligation assay with anti-IFIT5 mouse-derived antibodies and anti-protein G of the VHSV rabbit-derived antibodies, higher counts of RBC+ among VHSV-exposed cells were found (4). It should be added that IFIT proteins contain tetratricopeptide repeats and belong to the family of proteins that are strongly induced following production of interferon type I (IFN type I); moreover, as they are involved in transcription and translation regulation as well as replication of viruses, these proteins confirm a probable role of the IFIT5 in the antiviral response of RBCs to the VHSV infection (4). The studies of rainbow trout (*O. mykiss*) (4) suggest that the IFIT5 can participate in the antiviral process via binding VHSV RNA and limiting its replication. Expression and antiviral activity of the IFIT5, observed by Chico et al. (4), may not depend on interferon type I production, which seems to be of special interest. A similar lack of dependence, increased expression of antiviral genes, *ISG15* (interferon-stimulated gene 15), *mx, pkr* (RNA-activated protein kinase, also known as protein kinase R, PKR), and *ifit5*, with decreased IFN expression, have been demonstrated in the studies of rainbow trout (*O. mykiss*) erythrocytes exposed to glycoprotein G of the VHSV (26).

In the rainbow trout (*O. mykiss*), immune responses also involve shRBCs (shape-shifted RBCs) whose morphology has been changed owing to thermal stress in the *in vivo* environment (5). They are characterized by, e.g., a round shape, hemoglobin loss, and expression of new, IgM-like markers (5). These cells show the ability to inhibit VHSV infection as well as antiviral activity, which is manifested by increased expression of IL8, IL1, IFNγ, and NKEF. In addition, it has been demonstrated that shRBCs protect the TPS-2 (trout pronephric stroma-2) cells from the VHSV infection, which was shown by increased expression of IFNγ-activated *il1b, il6, il12, il15, tnfa* (TNFα gene), and *inos* (the gene of inducible nitric oxide synthase, iNOS) as well as of the IL8 encoding gene (5).

Involvement of erythrocytes in the defense against the VHVS has also been confirmed by the process of autophagy and the Nkl peptide associated with autophagolysosomes in erythrocytes, observed in a turbot (*Scophthalmus maximus*) (3).

### Piscine Orthoreovirus (PRV, PRV-1)

The PRV belongs to the family *Reoviridae*, subfamily *Spinareovirinae*, genus *Orthoreovirus*, and its genetic material is composed of dsRNA (double-stranded RNA). The genome of PRV consists of 10 segments: L1–3, M1–3, and S1–4. This group of viruses comprises three genotypes, PRV-1, 2, and 3, that infect the Atlantic salmon (*Salmo salar*), Chinook salmon (*Onchorhynchus tshawytscha*), Coho salmon (*Oncorhynchus kisutch*), rainbow trout (*O. mykiss*), and brown trout (*Salmo trutta*). The PRV-1 replication occurs in erythrocytes of the Atlantic salmon (*S. salar*), *in vivo* and *ex vivo*. The virus is widespread in the natural environment and associated with skeletal muscle inflammation (HSMI) in the farmed Atlantic salmon (*S. salar*) [The ICTV Report, https://talk.ictvonline.org/ictv-reports/ictv_online_report/, (27)].

It has been proved that erythrocytes of the farmed and wild Atlantic salmon (*S. salar*) are the main target cells for the PRV-1; the same refers to the PRV-2 which causes erythrocytic inclusion body syndrome in the Coho salmon (*O. kisutch*) and to the PRV-3 genotype which is associated with the HSMI-like disease in the rainbow trout (*O. mykiss*) and proliferative darkening syndrome in brown trout (*S. trutta*) (17, 27). PRV-1-infected RBCs of the Atlantic salmon (*S. salar*) induce expression of genes encoding antiviral immunity components, i.e., TLR3, RIG-I-like receptors, IFN-α, Mx, and PKR (17, 27). Induction of comparably wide gene range has been demonstrated based on the analysis of high-density oligonucleotide microarrays of PRV-infected erythrocytes of the Atlantic salmon (*S. salar*). This referred to increased expression of the *inf*α, *irf1* (interferon regulatory factor 1, IRF1), *mx, pkr*, and *rig-1* (retinoic acid–inducible gene 1) as well as the genes involved in antigen presenting by MHC type I molecules (19). HSMI-related PRV-1 viruses also induce expression of the *socs1* (suppressor of cytokine signaling 1) and *crfb4* (interleukin 10 receptor subunit beta) (19). However, it should be noted that *in vivo* and *ex vivo* susceptibility of the Atlantic salmon (*S. salar*) erythrocytes to the PRV infection is different (28) and probably results from differences in sensitivity of this virus to maturation-related immune activity of erythrocytes. It has been shown (17) that the total amount of RNA and synthesis of PRV proteins are higher in younger erythrocytes of Osteichthyes. The analysis of high-density oligonucleotide microarrays of PRV-infected RBCs of the Atlantic salmon (*S. salar*) also revealed a panel of genes with decreased expression compared with the pre-infection activity (19), including the *mmp9, mmp13a, mmp14* (matrix metallopeptidases, MMP), *ncf1* (neutrophil cytosol factor 1, NCF1), *cyba* (cytochrome b-245 alpha chain, also known as p22-phox), and others, such as the *csf1ra* (colony stimulating factor 1 receptor a) and *csf3r* (colony stimulating factor 3 receptor) which are involved in regulation of the inflammatory and immune processes. The authors of the research (19) conclude that such a wide scope of erythrocyte regulation potential indicates their multifunctionality in the process of immune response shaping, with the absence of this activity effects on gas transport and exchange.

### Rock Bream Iridovirus

RBIVs, as related, unclassified viruses, belong to the family *Iridoviridae*, subfamily *Alphairidovirinae*, genus *Megalocytivirus*. The genome of these viruses consists of dsDNA. The iridoviral infection is characterized by formation of IBCs (inclusion body-bearing cells) that are present in hematopoietic tissues, i.e., the spleen and anterior kidneys, as well as in the gills and the digestive tract. Among the fish species that are susceptible to these viral infections, e.g., *Siniperca chuatsi* (mandarin fish), *Pagrus major* (red seabream), or *Oplegnathus fasciatus* (rock bream, barred knifejaw, or striped beakfish) are mentioned (The ICTV Report, https://talk.ictvonline.org/ictv-reports/ictv_online_report/).

Based on the proteomic profiling of RBCs in the RBIV-infected rock bream (*O. fasciatus*), a higher ability of these cells to process and present the antigen by the MHC I with decreased expression of the gene *ISG15* has been demonstrated (16). Expression of this gene depends on IFN; it is involved in regulation of the antiviral response to various viruses, e.g., the IPNV in the Atlantic salmon (*S. salar*) and the GNNV (grouper nervous necrosis virus) in the orange-spotted grouper (*Epinephelus coioides*) (29). It has been documented (16, 29) that the immune response, related to the IFN and ISG15 protein activity, inhibits viral infections although in RBCs of the RBIV-infected rock bream (*O. fasciatus*), proteins associated with the ISG15 antiviral mechanism, such as the IRF3 (interferon regulatory factor 3), NUP35 (nucleoporin 35), and TRIM25 (tripartite motif containing 25), were decreased (16). Also, a higher activity of proteins related to the MHC I function, i.e., antigen processing and presenting, including TAP2 (transporter 2, ATP binding cassette subfamily B member) and proteasome subunits: 26S—PSMD11 (Proteasome 26S subunit, non-ATPase 11) and PSMD5 (Proteasome 26S subunit, non-ATPase 5) as well as PSMB6 (Proteasome subunit beta 6), PSMB3 (Proteasome subunit beta 3), and PSMB4 (Proteasome subunit beta 4), has been shown (16). RBC apoptosis plays an important role also in the mechanism of antiviral immunity and inhibition of RBIV replication in infected fish *O. fasciatus*. Proteomic analysis of these cells showed an increase in expression of many proteins associated with the apoptosis pathway: CASP6 (caspase 6), CASP9 (caspase 9), and FAS (Fas cell surface death receptor) (16). Most likely, through their activity related to processing and presentation of antigen involving MHC I molecules and the pathway associated with apoptosis, these cells induce the activity of cytotoxic T cells (CD8^+^) and the release of perforin and granzyme, which are the main factors of the mechanism of cytotoxicity and apoptosis of an infected and antigen-presenting cell in the context of MHC I (16).

### Infectious Salmon Anemia Virus

The ISAV belongs to the family *Orthomyxoviridae*, genus *Isavirus*. The genome of *Isavirus* viruses consists of eight independent negative-sense ssRNA segments. The genes of segments 1, 2, and 4 encode P proteins (79.9, 80.5, and 65.3 kDa) that form viral RdRp (RNA-dependent RNA polymerase), segment 3 encodes NP (nucleoprotein), segment 5 encodes F protein (type 1 membrane glycoprotein) (48.8 kDa), segment 6 encodes HE (type 1 membrane glycoprotein) (42.7 kDa), segment 7 encodes NS—non-structural protein and a protein of unknown function, and segment 8 encodes M1 (matrix protein 1) and RNA binding protein. The ISAV agglutinates erythrocytes of many fish species (The ICTV Report, https://talk.ictvonline.org/ictv-reports/ictv_online_report/).

The ISAV causes infectious salmon anemia (ISA) in the Atlantic salmon (*S. salar*) and is captured by erythrocytes (11). It induces an extensive immune response of these cells, which has been confirmed by testing mRNA levels of genes associated with the IFN type I pathway (IFN type I-induced), e.g., the genes encoding Mx, ISG15, and STAT1 (signal transducer and activator of transcription 1). In response to hemagglutination caused by a highly pathogenic ISAV isolate (NBISA01), i.e., in response to recognition of a viral HE protein (hemagglutinating and receptor-destroying glycoprotein) and fusion glycoprotein F as well as to engulfing of the virus during the endocytosis process (which is the basis of ISA pathogenic potential in the Atlantic salmon), a significantly high increase in the expression of IFN-α genes and slightly increased expression of the Mx, ISG15, STAT1, and PKZ (protein kinase containing Z-DNA binding domains, PKR-like) were observed, which is comparable with the expression seen in the infection caused by a less pathogenic ISAV (RPC/NB-04-085-1) whose hemagglutinating activity was only limited to virus adsorption without the next endocytosis stage, but such a response, according to researchers (11), may result from recognition of the viral HE protein during the process of hemagglutination and/or adsorption. It is interesting that the Atlantic salmon erythrocyte stimulation with Poly I:C, which is structurally similar to double-stranded RNA, did not cause induction of the IFN type I genes (11). In the view of presented data, it should be noted that pattern recognition receptors are involved, at different stages of the infectious process, in detection of various viral pathogen-associated molecular patterns, dsRNA, ssRNA, DNA, and glycoproteins, e.g., hemagglutinin (11).

### Infectious Pancreatic Necrosis Virus

The IPNV belongs to the family *Birnaviridae* and the genus *Aquabirnavirus*; its dsDNA genome consists of two linear segments (A and B). Segment A encodes the VP5 protein and preVP2—the VP2 precursor, VP4—called the NS protein (non-structural protein) in the salmonids, and VP3, while segment B (a smaller one) encodes VP1. The virus causes infectious pancreatic necrosis (IPN) in the salmonids, primarily in young brook trout (*Salvelinus fontinalis*), which is characterized by physiological impairment of this organ. The IPNV may also appear in other organs without disease manifestations (The ICTV Report, https://talk.ictvonline.org/ictv-reports/ictv_online_report/).

The INPV, non-infectious for RBCs of the rainbow trout (*O. mykiss*), induces antiviral immune activity of these cells (9), which was also observed in the case of infections caused by the ISAV (11) and PRV (17) in Atlantic salmon (*S. salar*). In *ex vivo* studies (9), increasingly higher, exposure time-dependent expression of the INF-1 (interferon type I) pathway-related genes (ISGs), i.e., *mx1*-3, *pkr, ifn1*, and *irf7* (interferon regulatory factor 7, IRF7), and increased expression of the *tlr3* have been demonstrated. The aforementioned alterations may suggest activation of the TLR3 pathway and increased production of IFN type I owing to the presence of viral dsRNA (9). It has also been shown that RBCs of Osteichthyes, although they are not susceptible to IPNV infection, may present an extensive antiviral response, similarly to the VHSV infection in the rainbow trout (*O. mykiss*) (6). In addition, IPNV-exposed erythrocytes manifested decreased levels of IL-1β, IL-8, and TNFα, which is most probably associated (as observed in the IPNV-infected CHSE-214 cell line, Chinook Salmon Embryo, ATCC CRL-1681) with loss/inhibition of the eIF2α (eukaryotic initiation factor 2α) activity, regulated in the process of phosphorylation (30). Thus, a different situation has been reported from that observed in the rainbow trout RBCs where the VHSV induced slightly increased eIF2α phosphorylation and, therefore, created a possibility of VHSV replication inhibition in RBCs of the trout (*O. mykiss*) (10).

## Antibacterial and Antifungal Activity of Erythrocytes in Fish

In addition to viruses, erythrocytes of Osteichthyes manifest their defense functions against bacteria (13) and fungi (18). The findings of research by Qin et al. (13) suggest that RBCs of a grass carp (*Ctenopharyngodon idella*) interact with such bacteria as *Staphylococcus aureus* and *Escherichia coli*. This is manifested by the adherence process and simultaneous, process-specific changes in morphology of erythrocytes, i.e., formation of a network of spicules that encompass microorganisms, and the engulfing ability in the phagocytosis process as well as the ability to activate a unique pathway of forming ROS via hemoglobin oxidation (13). It has been proved that RBC-released hemoglobin demonstrates a significant antibacterial activity, which is expressed by increased production of HbFe^IV^ (ferryl-Hb) and ROS; thus, it limits the survival of bacteria, up to 40% in the case of *E*. *coli* and up to 25% in the case of *S*. *aureus* and *Aeromonas hydrophila* (13). The phagocytic activity of RBCs has also been confirmed in the tests using FITC-labeled (fluorescein isothiocyanate) bacteria—*A. hydrophila* and *S. aureus* as well as GFP-labeled (green fluorescent protein) ones—*E*. *coli* (13).

In addition, the defense functions of erythrocytes in fish have been confirmed by the responses of these cells to the *Candida albicans* antigen (18). It has been shown that RBCs of the rainbow trout are capable of *C. albicans* binding and engulfing, however, with a lack of potential for further stages of phagocytosis i.e., the lysis of engulfed antigen molecule. As indicated by the authors (18), this may suggest that RBCs in fish do not have a mechanism and ability to kill an intracellular antigen in the oxygen-dependent and oxygen-independent processes. However, this research has shown another property of these cells, i.e., the ability of *C. albicans* antigen-loaded RBCs to stimulate defense and/or phagocytic activity of macrophages and to form rosettes with these cells, which ultimately, according to Passantino et al. (18), may lead to increased phagocytosis of *C. albicans* antigen by these cells. Also, it should be noted that phagocytosis by rosette-cocreating macrophages is more intensive compared with macrophages that are not involved in this reaction (18).

## Conclusions

Contrary to the common opinion and according to the previous studies, it is currently known that erythrocytes of Osteichthyes participate in innate and adaptive immune processes. The immune functions of these cells are manifested, among others, by transcription and translation of, e.g., viral proteins and the ability to cooperate with leukocytes and to produce cytokines. Moreover, RBCs of Osteichthyes demonstrate the efficient binding with a pathogen and capability of its engulfing in the processes of phagocytosis and intracellular destruction as well as the ability to generate ROS by extracellular hemoglobin. In these animals, in the face of the natural dominance of these mechanisms of non-specific immunity—more mature than specific immunity, it is important to highlight the possible evolutionary connection of nucleated erythrocytes in Osteichthyes and non-nucleated erythrocytes in mammals, which evolved mainly to ensure the fulfillment of oxygen needs of the organism while preserving immunomodulatory properties and the important role in the activation and/or stabilization of mechanisms of innate immunity as fish erythrocyte derivatives. The scope and scale of immune activity of erythrocytes in Osteichthyes are astonishing, particularly when they are assessed in terms of a large number of these cells in fish. Hence, it seems that even closer understanding of RBCs' immune functions in Osteichthyes and their ability to influence the components of innate and adaptive immunity may result in new possible ways of fighting with many viruses, bacteria, and fungi in these animals (6).

## Author Contributions

All authors listed have made a substantial, direct and intellectual contribution to the work, and approved it for publication.

## Conflict of Interest

The authors declare that the research was conducted in the absence of any commercial or financial relationships that could be construed as a potential conflict of interest.
